# Direct aorta implantation of microaxial blood pump via right anterior thoracotomy

**DOI:** 10.1016/j.xjtc.2023.04.003

**Published:** 2023-04-20

**Authors:** Kazuyoshi Takagi, Kosuke Saku, Takanori Kono, Yasuyuki Zaima, Yoshihisa Matsushima, Takehiro Homma, Tatsuhiro Shibata, Maki Otsuka, Michiko Yokomizo, Kensuke Ohshita, Yoshihiro Fukumoto, Eiki Tayama

**Affiliations:** a Division of Cardiovascular Surgery, Department of Surgery, Kurume University, School of Medicine, Kurume, Japan; b Division of Cardiovascular Medicine, Department of Internal Medicine, Kurume University, School of Medicine, Kurume, Japan; c Department of Anesthesiology, Kurume University, Kurume, Japan

**Figure:**
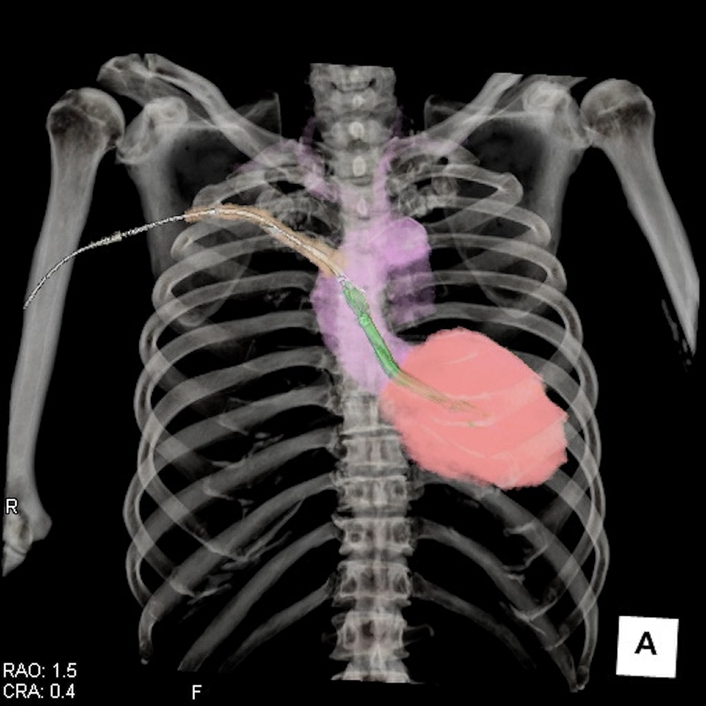
Position of Impella 5.5 direct aorta implant via right anterior mini-thoracotomy.

**Central Message**

Impella 5.5 direct aorta implantation via right anterior thoracotomy is a useful technique for patients with limited peripheral vascular size and can be placed similarly to the axillary approach.

The Impella 5.5 with SmartAssist system (Im5.5; Abiomed), is the latest-generation microaxial mechanical circulatory support (MCS) with longer support duration and greater pump flow. Although Im5.5 was designed for transaxillary implantation, the direct ascending aorta (DA) and transinnominate implantation via sternotomy have been employed in pediatric patients, patients with small-sized (<7 mm) axillary arteries, and in patients with peripheral vascular disease.^1^, ^2^, ^3^ Herein, we present the DA-Im5.5 implant via right anterior thoracotomy (RAT). Written informed consent was obtained from the patient for this report and any accompanying images (Kurume University institutional review board number 21001, April 20, 2021).

## Case Report

**Figure 1:**
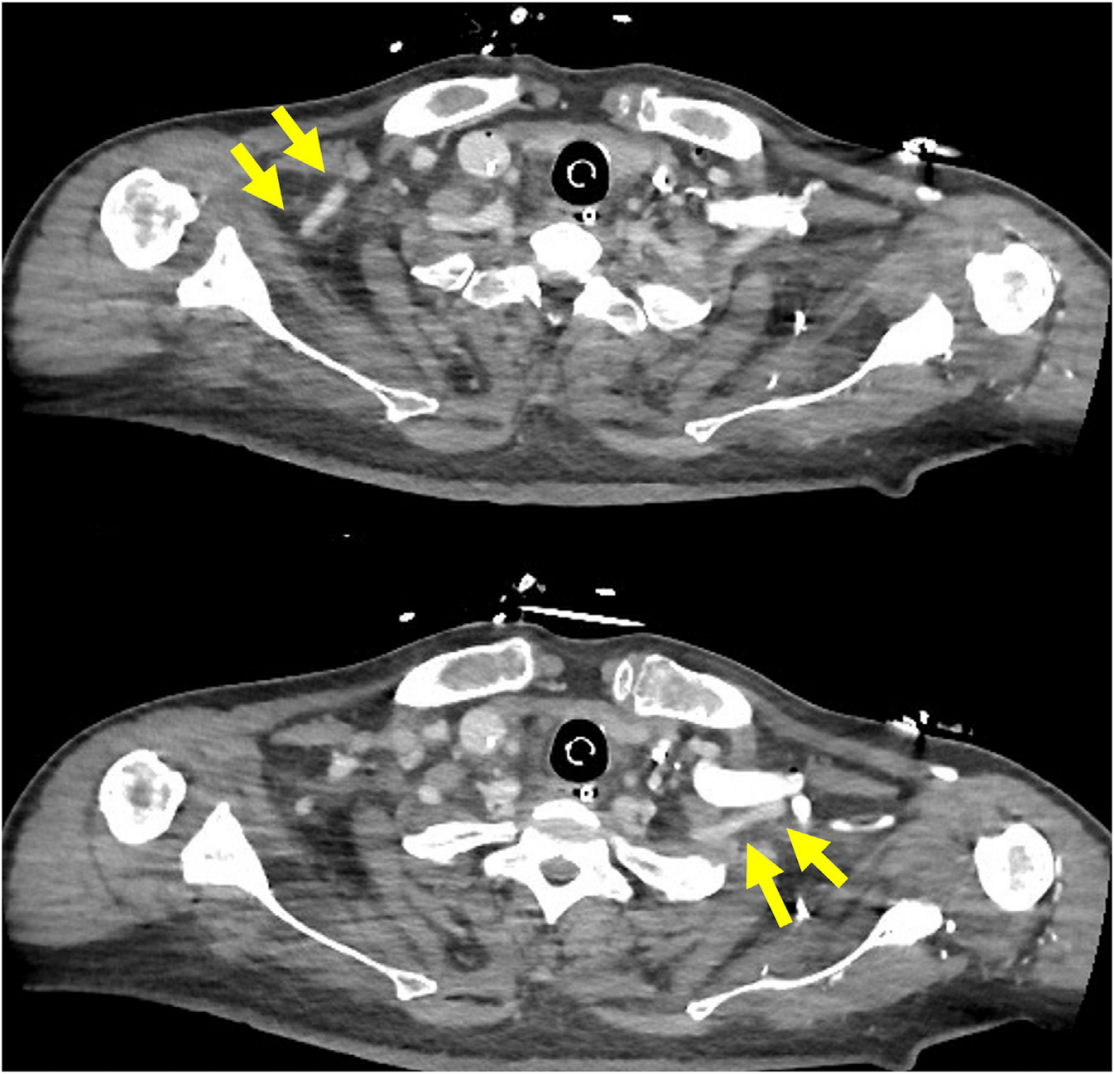
Preoperative computed tomography (*CT*) shows bilateral axillary artery measuring less than 5.5 mm (*yellow arrows*).

A 65-year-old man (body surface area: 1.9 m^2^) with a history of percutaneous coronary intervention for anterior wall myocardial infarction was hospitalized due to dyspnea and leg edema. Echocardiography showed a left ventricular ejection fraction of 15%. Cardiac catheter examination revealed pulmonary artery wedge pressure of 40 mm Hg and a low cardiac index of 1.26 L/min/m^2^. He was on dobutamine at 3 μg/kg/min and norepinephrine at 0.12 μg/kg/min with arterial pressure of 95/72 mm Hg and serum lactate level of 3.8 mmol/L. He was diagnosed with cardiogenic shock due to ischemic cardiomyopathy but was not a candidate for a heart transplant and ventricular assist device in Japan. We considered the longer Im5.5 support required for the recovery and establishing appropriate medication. However, bilateral axillary arteries were measured 5.0 to 5.5 mm using computed tomography (Figure 1). We performed DA-Im5.5 via RAT.

**Figure 2:**
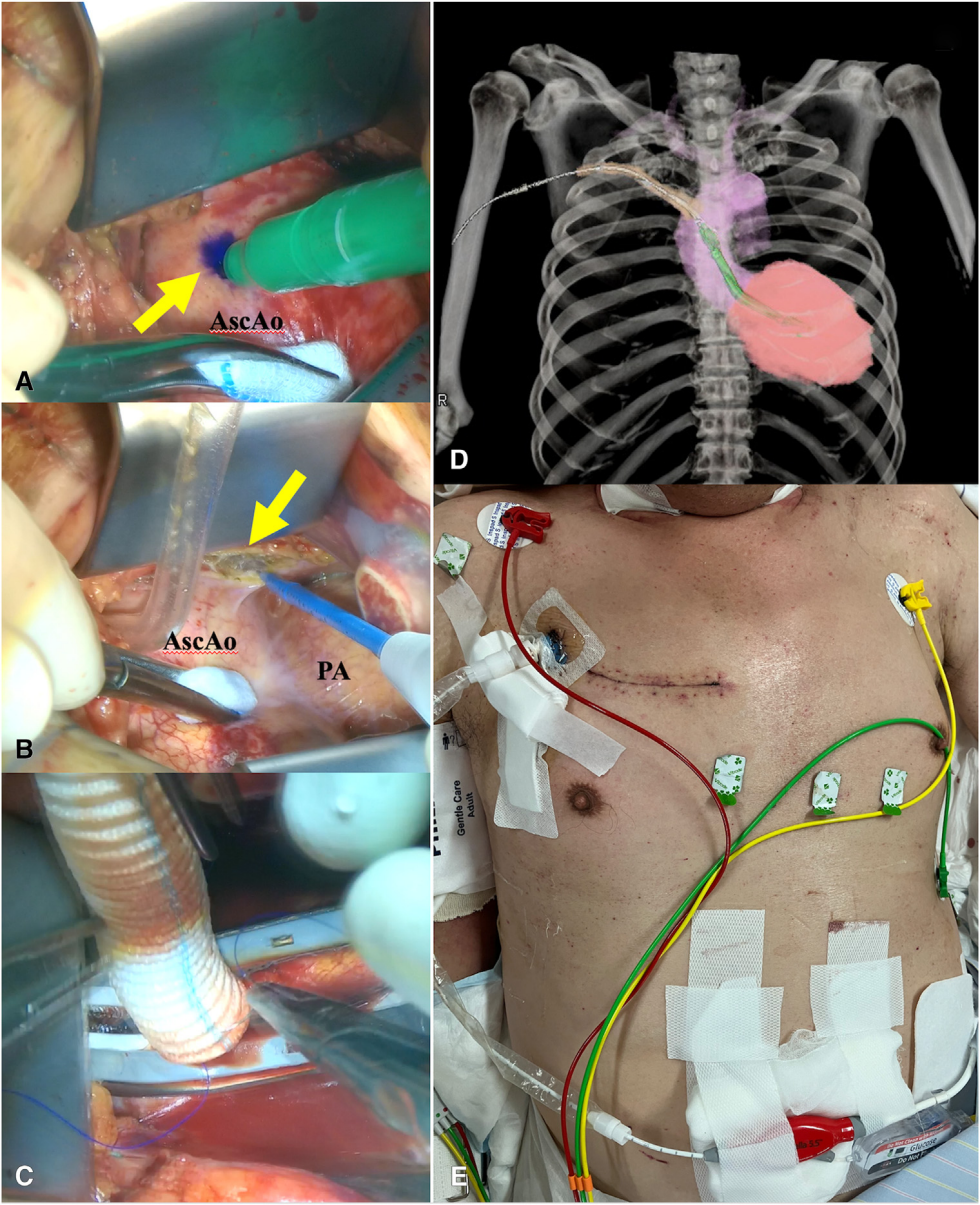
A, *Yellow arrow* shows the anastomosis site 1 cm below the origin of innominate artery. B, *Yellow arrow* shows the resected reflection of pericardium. C, End-to-side anastomosis performed using a partial clamp at the center of the wound. D, Postoperative computed tomography showing adequate position and gentle direction of Impella 5.5 in ascending aorta and left ventricle. E, Impella 5.5 was placed similar to the axillary approach. *AscAo*, Ascending Aorta; *PA*, pulmonary artery.

We made a 6-cm transverse skin incision in the second intercostal space adjacent to the sternum under general anesthesia with bilateral lung ventilation (Video 1). The third rib was cut medially on the costal cartilage. The pericardium was opened, and 7 pericardial stay sutures were placed. A graft should be anastomosed to the distal ascending aorta (AscAo), at least 7 cm above the aortic valve (Figure 2, *A*). Reflection of the pericardium was resected around distal AscAo to easily deliver the anastomosis site toward the center of wound (Figure 2, *B*). The second rib was disarticulated for clamp placement. After systemic heparinization, a 9-mm J-graft (J Graft; Japan Lifeline) was sewn end-to-side to the AscAo using 5-0 polypropylene running suture after partial clamping (Figure 2, *C*). The graft was clamped above the anastomosis. A separate 2-cm skin incision was made in the subclavicular space. The graft was tunneled through the first intercostal space in a gentle upward direction (Figure 2, *D*). Im5.5 was introduced using the standard insertion technique under fluoroscopic and transesophageal echocardiography guidance. The graft was trimmed to the skin level and pushed under the wound. The second and third ribs were fixed using a mesh plate (Grand Fix; Abbott Japan) and steel wires. Im5.5 position was stable and similar to axillary implantation (Figure 2, *E*). Although the patient received slight sedation using dexmedetomidine hydrochloride during Im5.5 support, an additional pain regimen was not needed. Two weeks after surgery, Im5.5 was removed under local anesthesia in the intensive care unit. The surgical incision was opened, Im5.5 was withdrawn, and graft was ligated into the right thoracic cavity.

## Comment

A greater flow, the lack of a pigtail catheter to reduce interference with the valvular chordae and thrombus, and the low incidence of hemolysis are specific improvements of Im5.5. Im5.5 is a useful temporary MCS for weeks as the bridge to recovery, ventricular assist device, and heart transplant for various cardiogenic shock,^4^ except for patients with severe right ventricular failure, malignant ventricular arrhythmias, and hemorrhagic disorders.

Although an alternative Im5.5 implantation via sternotomy is easily performed,^1^, ^2^, ^3^ DA-Im5.5 via RAT is useful for avoiding fatal complications during prolonged support. Thromboembolism induced by the Impella occasionally occurred during removal, especially after prolonged support. The transinnominate approach has a greater risk of cerebral infarction than the DA-Im5.5 approach. Late graft infection occurred in 10.9% of patients with Impella 5.0/5.5 via axillary, and most of them required explantation of the infected graft.^5^ Sufficient distance and exists of muscular and soft tissue between the insertion site and mediastinal space is another advantage to preventing the progression of graft infection to the deep surgical site. If necessary, the infected graft can be removed through the same RAT incision. Early mobilization, preserved right ventricular function, and the lowest potential for catheter migration due to a similar position to axillary implantation may be potential benefits.^2^

Inadequate access to AscAo is a potential risk, and the short length of AscAo, atherosclerosis, and porcelain aorta are contraindications of this approach. Therefore, detailed preoperative planning using 3-dimensional computed tomography should be performed.

We recommend disarticulation of ribs to obtain a surgical space and safety. Reliable rib fixation using a mesh plate and wire is useful to reduce postoperative pain.

## Supplementary Data

Video 1 Video shows the technique for anastomosing a graft to the ascending aorta (AscAo) with a partial clamp via right anterior minithoracotomy. A 6-cm transverse skin incision was made in the second intercostal space. The third rib was disarticulated and the intercostal muscle above the fourth rib was resected 4 cm to push down the third rib under the fourth rib. The pericardium was opened, and 7 pericardial stay sutures were placed. The reflection of pericardium was resected around the AscAo. We decided that the anastomosis site was 1 cm below the origin of the innominate artery. The second rib was disarticulated for clamp placement. A 9-mm J-graft was sewn end-to-side to the AscAo using a 5-0 polypropylene running suture. A separate 2-cm skin incision was made in the subclavicular space. The graft was tunneled through the first intercostal space in a gentle upward direction. Video available at: https://www.jtcvs.org/article/S2666-2507(23)00125-6/fulltext.

## References

[1] Anderson M., Smith D., Kane P., Lee R., Khalpey Z., Williams J. (2021). Impella 5.5 direct aortic implant and explant techniques. Ann Thorac Surg.

[2] Bertolin S., Maj G., Cavozza C., Cardinale A., Pullara A., Audo A. (2022). Impella 5.0/5.5 implantation via innominate artery: further expanding the opportunities for temporary mechanical circulatory support. J Clin Med.

[3] Bouhout I., Nguyen S.N., Barry O.M., Bacha E.A., Goldstone A.B. (2022). Transinnominate Impella 5.5 insertion as a bridge to transplantation in a pediatric patient in refractory cardiogenic shock. JTCVS Techniques.

[4] Rock J.R., Kos C.A., Lemaire A., Ikegami H., Russo M.J., Moin D. (2022). Single center first year experience and outcomes with Impella 5.5 left ventricular assist device. J Cardiothorac Surg.

[5] Lewin D., Nersesian G., Lanmüller F., Schoenrath F., Falk V., Potapov E.V. (2023). Complications related to the access site after transaxillary implantation of a microaxial left ventricular assist device. J Heart Lung Transplant.

