# Supplementary figures and images for: Direct aorta implantation of microaxial blood pump via right anterior thoracotomy
Source: JTCVS Tech. 2023 Apr 20;19:64–7. doi: 10.1016/j.xjtc.2023.04.003 (PMC10268493; doi:10.1016/j.xjtc.2023.04.003)

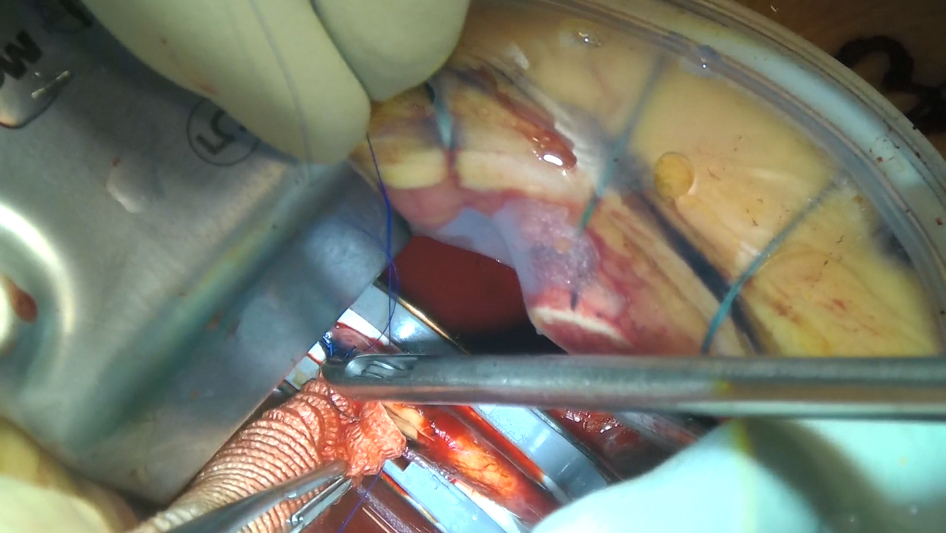

Supplement: Video 1 — Video shows the technique for anastomosing a graft to the ascending aorta (AscAo) with a partial clamp via right anterior minithoracotomy. A 6-cm transverse skin incision was made in the second intercostal space. The third rib was disarticulated and the intercostal muscle above the fourth rib was resected 4 cm to push down the third rib under the fourth rib. The pericardium was opened, and 7 pericardial stay sutures were placed. The reflection of pericardium was resected around the AscAo. We decided that the anastomosis site was 1 cm below the origin of the innominate artery. The second rib was disarticulated for clamp placement. A 9-mm J-graft was sewn end-to-side to the AscAo using a 5-0 polypropylene running suture. A separate 2-cm skin incision was made in the subclavicular space. The graft was tunneled through the first intercostal space in a gentle upward direction. Video available at: https://www.jtcvs.org/article/S2666-2507(23)00125-6/fulltext. [file fx2.jpg]
